# Energy and Momentum Distribution of Surface Plasmon-Induced Hot Carriers Isolated *via* Spatiotemporal Separation

**DOI:** 10.1021/acsnano.1c06586

**Published:** 2021-12-01

**Authors:** Michael Hartelt, Pavel N. Terekhin, Tobias Eul, Anna-Katharina Mahro, Benjamin Frisch, Eva Prinz, Baerbel Rethfeld, Benjamin Stadtmüller, Martin Aeschlimann

**Affiliations:** † Department of Physics and Research Center OPTIMAS,TU Kaiserslautern, Erwin-Schrödinger-Straße 46, 67663 Kaiserslautern, Germany; ‡ Institute of Physics, Johannes Gutenberg University Mainz, Staudingerweg 7, 55128 Mainz, Germany

**Keywords:** plasmon-induced hot carriers, surface plasmon polariton, PEEM, 2-photon photoemission, momentum microscopy

## Abstract

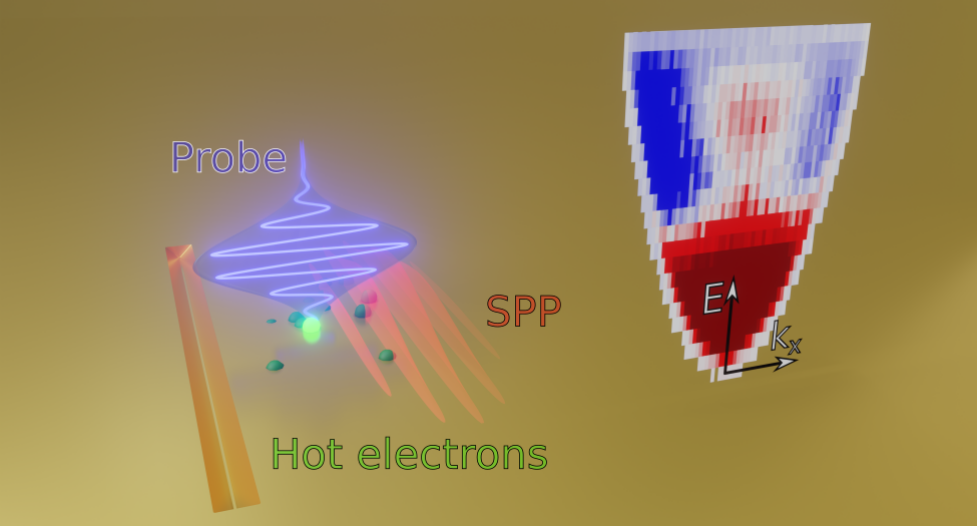

Understanding the differences between photon-induced and plasmon-induced hot electrons is essential for the construction of devices for plasmonic energy conversion. The mechanism of the plasmonic enhancement in photochemistry, photocatalysis, and light-harvesting and especially the role of hot carriers is still heavily discussed. The question remains, if plasmon-induced and photon-induced hot carriers are fundamentally different or if plasmonic enhancement is only an effect of field concentration producing these carriers in greater numbers. For the bulk plasmon resonance, a fundamental difference is known, yet for the technologically important surface plasmons, this is far from being settled. The direct imaging of surface plasmon-induced hot carriers could provide essential insight, but the separation of the influence of driving laser, field-enhancement, and fundamental plasmon decay has proven to be difficult. Here, we present an approach using a two-color femtosecond pump–probe scheme in time-resolved 2-photon-photoemission (tr-2PPE), supported by a theoretical analysis of the light and plasmon energy flow. We separate the energy and momentum distribution of the plasmon-induced hot electrons from that of photoexcited electrons by following the spatial evolution of photoemitted electrons with energy-resolved photoemission electron microscopy (PEEM) and momentum microscopy during the propagation of a surface plasmon polariton (SPP) pulse along a gold surface. With this scheme, we realize a direct experimental access to plasmon-induced hot electrons. We find a plasmonic enhancement toward high excitation energies and small in-plane momenta, which suggests a fundamentally different mechanism of hot electron generation, as previously unknown for surface plasmons.

## Introduction

The world’s vital demand for clean energy poses a huge challenge for fundamental and application-oriented research to devise new and sustainable concepts to convert solar power into electric and chemical energy. One key perspective to enhance the efficiency of light-to-carrier conversion processes is plasmon technology. Despite the great potential of plasmon-induced hot carriers for photovoltaics and photochemistry,^1−3^ their precise role in the enhancement of the efficiency of these processes is still heavily discussed.^1,4,5^ Fundamental questions remain on how plasmon-induced hot carriers are generated, how they dissipate energy and momentum, and how the underlying mechanisms come into play in plasmonic energy conversion processes. Many theoretical studies have been conducted in the last years to address these questions.^6−17^ However, it is essentially unresolved if plasmonic enhancement is simply due to the field-enhancement of light at the surface of a metal, or if there is a more fundamental difference between plasmon-induced and photon-induced hot carriers.

For the bulk plasmon resonance, in the ϵ near zero range at the plasma frequency ω_p_, a fundamental difference has long been known from theoretical studies^18^ and was more recently shown in linear^19,20^ and nonlinear^21,22^ photoemission experiments: Bulk plasmons selectively excite electrons from occupied states close to the Fermi energy *E*_F_ of the metal. This results in a peak in the photoemission spectrum at *E* – *E*_F_ = *ℏω*_p_ (or 2*ℏω*_p_ in the nonlinear case). This energy-selective emission strongly contradicts the expectation for the electron and hole distributions in conventional photoexcitation, which are governed by the density of states (DOS) of the material. The effect was referred to as non-Einsteinian photoemission^21−23^ due to its fixed energetic position for all photon energies.

In energy harvesting applications, often energy thresholds have to be overcome for efficient charge separation or driving a chemical reaction. Therefore, a preferential high-energetic electron excitation, as observed for bulk plasmons, could hold great potential. This would require that a similar effect also exists for surface plasmons, which can be excited at optical frequencies and concentrate the energy density at the metal surface, where electrons can be transferred across a functional interface or they can be excited directly *via* chemical interface damping.^17,24^ A theoretical model for the bulk case by Novko *et al*. suggests that a similar plasmonic decay for surface plasmons might be possible.^23^ Although some experiments at nanostructure interfaces show an enhancement for high-energy electrons,^25−27^ a clear identification of the microscopic mechanisms has not yet been possible due to the complex interplay of various experimental parameters (field enhancement, interface effects, laser field, *etc.*)

Conventionally, plasmonic excitations and their decay are investigated experimentally using optical spectroscopy techniques.^28^ In such measurements, different decay channels can be quantified by the systematic variation of parameters.^29^ The particularly intriguing hot electrons can be addressed, at least indirectly, *via* time-resolved spectroscopy techniques,^30−32^ or in prototypical plasmonic devices by measuring photocurrents transmitted across Schottky-type interfaces.^33,34^

A more direct access to the excited electron (”hot carrier”) dynamics on the femtosecond time scale and in the single-particle limit can be gained by the time-resolved 2-photon-photoemission (tr-2PPE) technique.^35−39^ This method has been established for many years for the case of optical rather than plasmonic excitation, even in the context of hot carrier assisted photochemistry.^40^ On the contrary, plasmonic fields themselves have been successfully imaged with time-resolved photoemission electron microscopy (tr-PEEM),^41^ both for localized^42−44^ and propagating^44−46^ surface plasmons (LSP and SPP, respectively). In this context, the photoelectrons were used merely as an experimental observable for the plasmon field. Combining these two approaches of photoemission allows for imaging plasmon-induced hot carriers on the femtosecond and nanometer scale. However, the progress in this direction has been slow, as the question about the microscopic mechanism of surface plasmon damping arose two decades ago from early photoemission experiments on plasmonic samples.^47^ The separation of plasmon and electron dynamics was limited to describing the plasmon as a modified electromagnetic field in the vicinity of the surface,^48^ known as the plasmonic near-field.

Examining recent photoemission experiments with dominant surface-plasmon fields,^25,27,49−52^ it remains difficult to disentangle the fundamental plasmon decay from the multitude of physical effects that contribute to the observed nonlinear signals, including the influence of the driving laser. A temporal separation of the material response induced by the exciting laser pulse or the plasmon could in principle be achieved for experimental conditions for which the pulse duration is much shorter than the lifetime of an LSP at a nanostructure.^53,54^ But, even in those cases, experimental access to the fundamental difference between plasmonic and photonic electron excitation is denied by the inability to obtain respective photoemission signals with exactly comparable conditions, *e*.*g*., at a nanostructure with given shape and material, the plasmonic signal is easily measured but the photonic counterpart cannot be acquired without losing comparability. On a plane metal surface, where comparability is given, photoemission with contribution of an unfocused SPP wave is common in tr-PEEM, but its imaging requires the interference with a probe laser pulse.^46^ When the plasmon and the probe laser field coexist in space and time, a manifold of possible transition pathways with arbitrary, indistinguishable contributions of the two fields takes part in photoexcitation into a state of given energy.^55^ This obscures the experimental access to the plasmon-induced hot electrons sought for.

In this paper, we employ two-color time- and energy-resolved PEEM experiments in real and momentum space to directly image plasmon-induced hot carriers. We demonstrate the separation of the photoelectrons generated in the process, in which first a plasmon decays by producing an excited (hot) electron, which is then photoemitted by a photon of double the energy. To achieve this, we use the spatiotemporal dynamics of a propagating SPP plane wave pulse. We perform a theoretical analysis of the flow of SPP energy density to separate the different contributing terms of SPP and laser analytically. Our method provides a direct imaging of plasmon-induced hot electrons, with sensitivity to the time, space, momentum, and energy domains.

## Results and Discussion

### Spatiotemporal Separation Scheme

**Figure 1:**
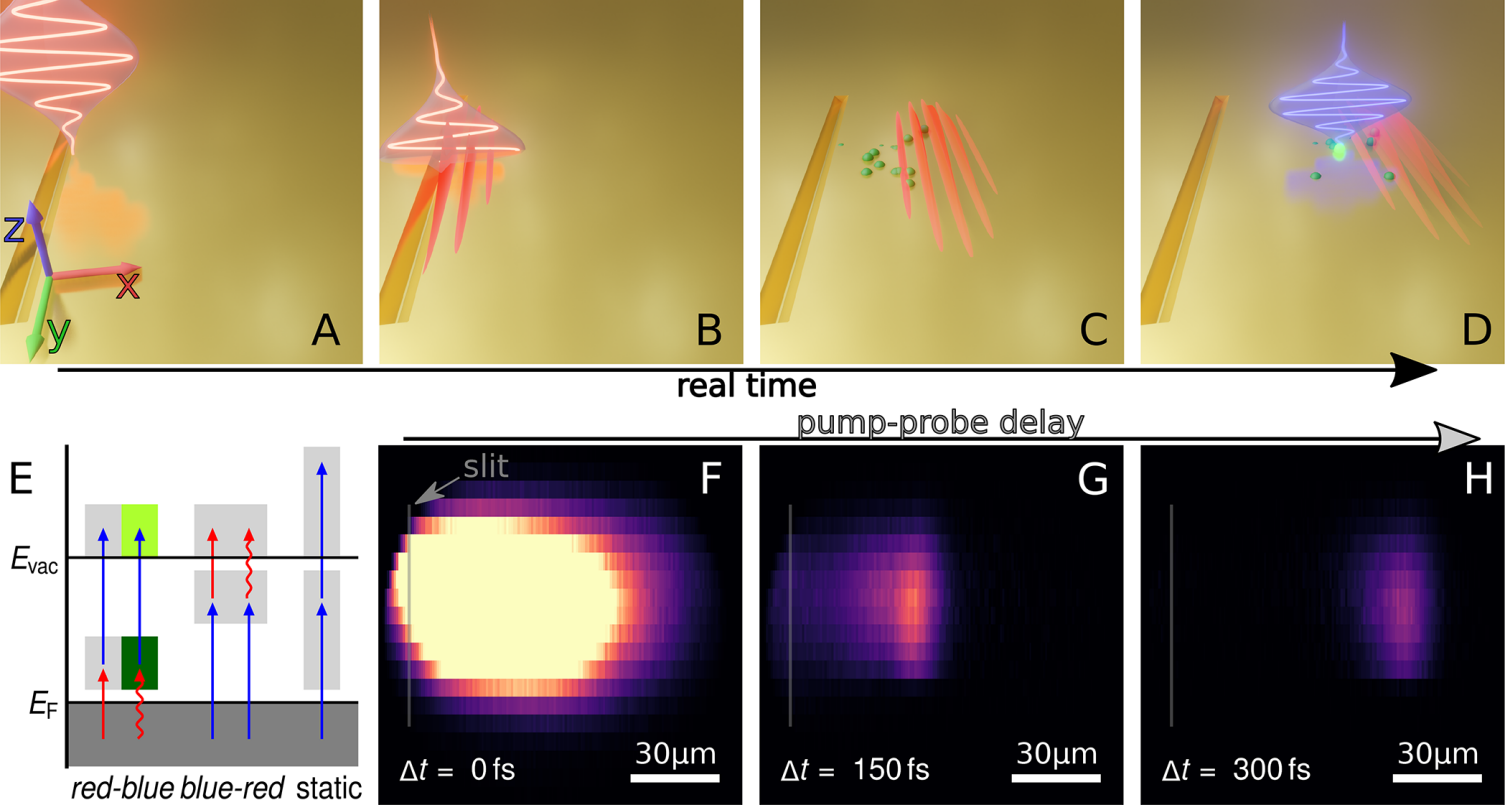
Scheme of spatiotemporal separation. (A) Pump pulse (red) arrives at the coupling slit etched into the Au surface. (B) SPP pulse is excited and interacts with the remaining pump field during the pulse duration. (C) Plasmon pulse propagates along the surface, exciting hot electrons (green balls) as it is damped through internal decay. (D) Plasmon-excited hot electrons are photoemitted by a probe pulse (blue). A photoelectron (highlighted green ball) is produced, which is subsequently extracted and detected by the PEEM optics. The frames were taken from an animated video of the sequence, provided in the Supporting Information. (E) Energy diagram of possible 2PPE channels. Transitions are marked as arrows representing the driving force, straight red for the pump pulse, curly red for the SPP, and straight blue for the probe pulse. The relevant energy ranges of excited and photoemitted states are marked in green for the plasmon-induced hot carriers under investigation, or in light gray, respectively, for the other channels. The dark gray area represents occupied electronic states up to *E*_F_. The green highlighted channel is isolated by means of the spatiotemporal separation scheme. (F–H) PEEM images of time-dependent photoemission signal (*Y*_source_), which is dominated by photoemitted electrons from the red–blue channel. The PEEM images were integrated in an excited state energy range *E* – *E*_F_ = 1.45–1.55 eV, and the snapshots were extracted for pump–probe delays of Δ*t* = 0, 150, and 300 fs, respectively. The gray line represents the position of the excitation slit at *x* = 0 μm. A video of the full time-resolved PEEM series is available in the Supporting Information.

Our experimental scheme to separate the photon- and plasmon-induced hot carriers is sketched in Figure 1. An ultrashort SPP pulse is excited at a coupling slit, engraved into a gold layer, oriented along the *y*-direction at *x* = 0 μm. The red pump pulse (τ_pump_ ≈ 23 fs, λ_center_ = 800 nm ⇒ *hν* = 1.55 eV) is focused on the coupling structure under near-normal incidence, with linear polarization in *x*-direction, perpendicular to the slit. The structure constitutes a break of translation symmetry, providing additional momentum, which allows for the coupling of light into the SPP mode. In that way, an SPP pulse with a plane wavefront is launched, which then propagates along the Au–vacuum interface in positive *x*-direction (a corresponding pulse propagating in negative *x*-direction is also launched from the slit, but it is outside of the detection area).

The damping of the SPP is dominated by the internal decay in the metal, producing electron–hole pairs, because of the nature of the SPP being a dark mode that cannot decay into the far-field without a break of symmetry at the surface. In this way, the SPP pulse acts as a propagating plasmonic source of hot electrons.

We probed the hot electron population by a time-delayed irradiation of the sample surface with a blue laser pulse (τ_probe_ ≲61 fs, λ_center_ = 400 nm ⇒ *hν* = 3.1 eV), generated by second harmonic generation from a split-off part of the fundamental output of the laser light source. Using a submonolayer of Cesium evaporated onto the sample, the work function of the Au surface was reduced to Φ ≈ 3.4 eV. In this way, the hot electrons with an excitation energy of *E* – *E*_F_ ≥ 0.3 eV can be photoemitted by absorbing a photon from the probe pulse. A photoemission electron microscope (PEEM) was used to image these electrons from the sample to a detector that is sensitive to their position and energy (see the Methods section). In this way, a 4-dimensional data set of photoelectron yield *Y*(*x*, *y*, *E*, Δ*t*) was recorded by scanning the delay times between the pump and probe pulses and acquiring *x*–*y*- and energy-resolved PEEM images for each time step.

With our experimental conditions (see the Methods section), we are well in the single-particle regime, *i*.*e*., the density of optically excited electrons is too low to lead to any interactions between excited carriers. The optically excited electrons can only interact with (cold) electrons of the Fermi sea. No significant transient excitation of the phonon system out of equilibrium is induced.

The possible excitation channels that contribute to the measured photoelectron yield are shown in Figure 1E. In the data set, the relative yield of the static 2PPE by the probe pulse (”static” channel) can be referenced and subtracted, and the dynamic signal with contribution of excitations with 1.55 eV, namely, from the pump or SPP pulses, remains (Figure 1F–H). For this signal, there are two participating channels, which differ in the temporal order of the two sequential excitation steps. Apart from the previously outlined red–blue channel, in which the excited electron from the red pump pulse or SPP is photoemitted by the blue probe, the reversed order is also possible, in which the blue probe laser excites an electron, which is then photoemitted by the red pump laser or SPP (blue–red channel). The intermediate state of the blue–red channel is located at an energy 1.55 eV higher than that of the red–blue channel though. This state has an about 10 times shorter inelastic lifetime,^39^ following essentially the well-known relation *T*_1_ ∝ (*E* – *E*_F_)^−2^ from Fermi liquid theory. Neglecting band structure effects, the 2PPE yield scales at least linearly with *T*_1_; therefore, the red–blue channel dominates the signal (the exact scaling of the yield with *T*_1_ depends on dephasing of the coherences along the excitation pathway).

The further isolation of the contribution of the plasmon-induced hot carriers from those excited directly by the pump laser was performed using the spatiotemporal signature of their source, namely, the SPP pulse, which propagates along the surface of the sample on a femtosecond time scale. To characterize the spatiotemporal characteristics of this source as a reference for the following evaluation of the photoemission data, we analytically calculated the energy density flow of the time-dependent electromagnetic field in a model system that emulates the conditions in the experiment but without a probe laser pulse (see the Methods section).

We analytically derived the absorbed energy density rate by solving Maxwell’s equations for the laser and SPP fields. This calculation describes the laser energy absorption and defines the spatiotemporal characteristics of the available energy for the excitation of hot electrons. We showed in ref (56) that it contains different interference contributions (*laser* × *laser*, *laser* × *SPP*, and *SPP* × *SPP*). Each of these terms contributes to the first step of the red–blue photoemission channel in Figure 1E, producing the hot electrons in the intermediate state of the process. The first term, *laser* × *laser*, has no plasmonic contribution and is responsible for purely photon-induced electrons. The second term, *laser* × *SPP*, arises from the interference of the laser and SPP fields. We showed in ref (55) with a one-color phase-resolved PEEM experiment that this term produces mixed contributions in the photoemission process where even entangled quantum pathways take part, in which both fields participate in an indistinguishable manner. This corresponds to a mixing between the two red–blue channels displayed in Figure 1E. Therefore, a hot electron produced by the *laser* × *SPP* term cannot be clearly classified as photon-induced or plasmon-induced. However, the third term, *SPP* × *SPP*, describes the propagation and decay of the SPP pulse. This term is the origin of purely plasmon-induced hot carriers. A detailed description of the calculation and the resulting energy density rate in space and time is given in our previous work.^56^

**Figure 2:**
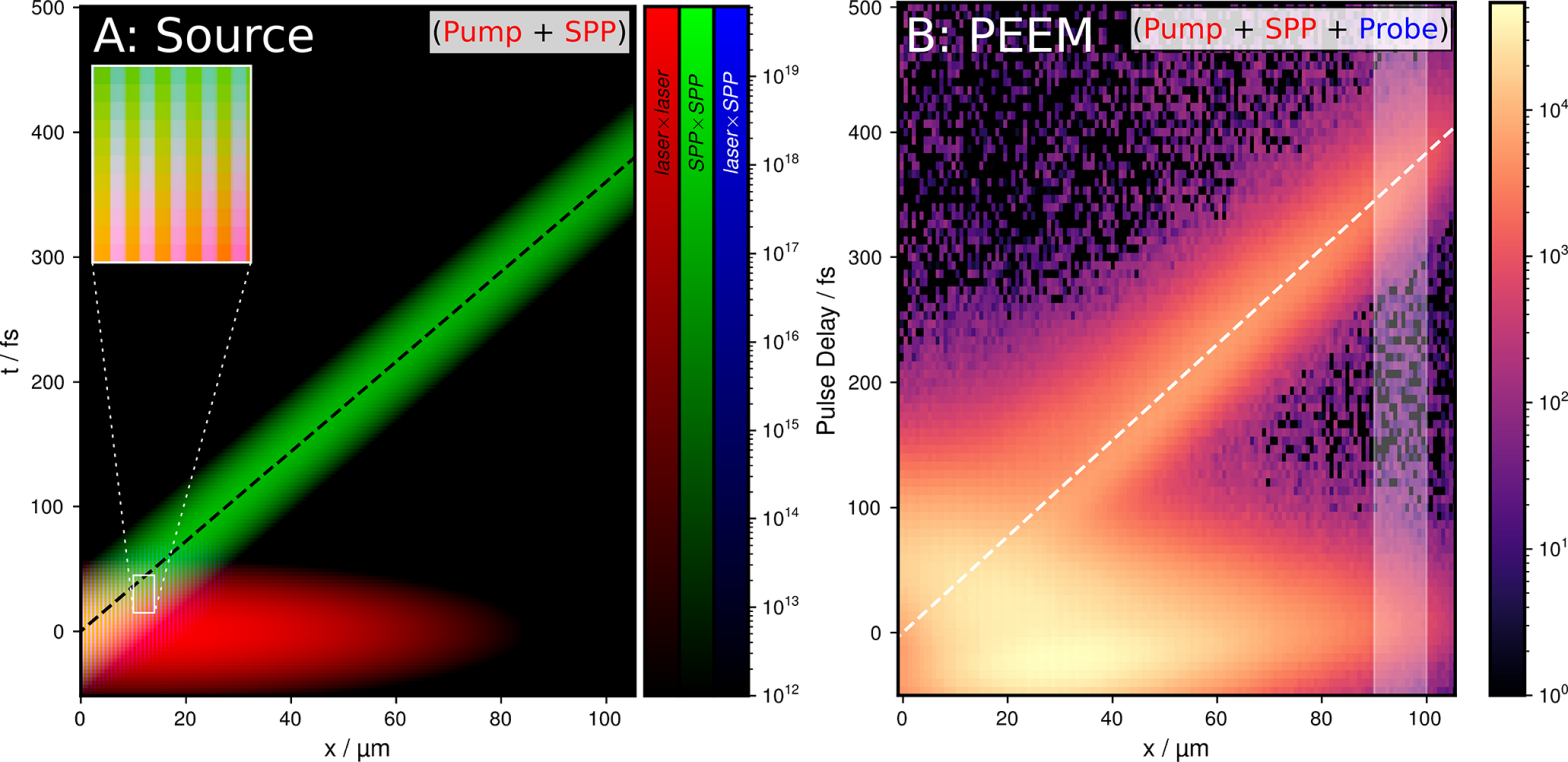
(A) Time-dependent energy density rate of the electromagnetic field contributions at the surface, for a coupling structure at *x* = 0 μm irradiated at normal incidence with a pump pulse centered at *t* = 0 fs. The three contributing terms, *laser* × *laser*, *SPP* × *SPP*, and *laser* × *SPP* are encoded in the red, green, and blue color channels of the image, with each colorbar given in J s^–1^ m^–3^. The additive color mixing leads to additional color shades where more than one field is present at the same time and position, which leads to yellow pixels for *laser* × *laser* and *SPP* × *SPP* and more blue-shaded colors for all three components. The velocity of an SPP, *v*_SPP_, excited with λ = 800 nm is plotted as a dashed black line. Plots of the single components are available in the Supporting Information. The inset is a zoom of *x* = 10–14 μm and *t* = 15–45 fs, where all three components are present and the contribution of the interference term *laser* × *SPP* is visible as blue-shaded stripes. (B) Measured tr-PEEM signal of the spatiotemporal signature of hot electrons, *Y*_source_, with the colorbar given in counts, at an excitation energy of 1.45–1.55 eV for an integration range of *y* = 55–79 μm. The static background *Y*_static_ was subtracted. The perceived velocity of the SPP pulse *v*_perceived_ (see the Methods section) is plotted as a white dashed line. The white shaded region represents the *x* integration range for Figure 3.

The spatiotemporal dependence of the energy density rate at the interface between Au and vacuum is shown in Figure 2A, with the three separate contributing terms encoded in the red, green, and blue color channels, representing *laser* × *laser*, *SPP* × *SPP*, and *laser* × *SPP*, respectively. The pump pulse (red channel, *laser* × *laser* term) is visible as a nonpropagating signal around *t* = 0 fs. The SPP (green channel, *SPP* × *SPP* term) appears as a propagating trace in positive *x*-direction until well past the irradiation time. The interference term (blue channel, *laser* × *SPP* term) is present only where the two fields overlap in space and time, leading to a periodic variation from yellow to blue-shaded colors (inset in Figure 2A).

The dashed black line in Figure 2A represents the SPP group velocity. This velocity can be derived from the SPP dispersion relation as
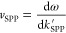
1where *k*_SPP_^′^ is the real part of the SPP wave vector
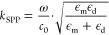
2where ω is the laser angular frequency, *c*_0_ is the speed of light, and ϵ_m_ and ϵ_d_ are the dielectric functions of the metal and dielectric half-spaces, respectively.

### PEEM Results

In the time-dependent energy density rate in Figure 2A, the laser and SPP contributions are clearly separated in time at larger distances from the excitation edge (*x* ≳ 40 μm). During the pulse duration τ_pump_, when the pump pulse impinges on the sample around *t* = 0 fs, the *laser* × *laser* term dominates. An electron that is photoemitted at this stage close to the excitation edge cannot be attributed unambiguously to either photonic or plasmonic excitation. In contrast, after the pulse has faded for *t* > τ_pump_, the situation is different: The SPP pulse still propagates along the surface as a moving source, producing hot electrons along its way. An electron that is photoemitted and detected during this stage might still remain from a photonic excitation, but especially for higher energies where electron lifetimes are as short as only a few tens of femtoseconds, the SPP pulse is the predominant source of hot electrons.

The experimental PEEM data can be analyzed in terms of the same space-time characteristics to assign types of origin to the detected electrons. From the acquired 4D data set of photoemission yield *Y*(*x*, *y*, *E*, Δ*t*), the contribution caused by the calculated dynamic source, *Y*_source_, was isolated by subtracting the static (delay-independent) 2PPE yield:
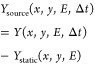
3

The static yield *Y*_static_ is derived from the relative signal before the two pulses overlap, by averaging the measured count values for the pulse delays in the range of Δ*t* < – 200 fs. The only delay-independent 2PPE channel (static in Figure 1E) is the two-photon contribution from the probe pulse (blue–blue). It is the dominant contribution to *Y*_static_. Additionally, a three-photon contribution from the pump pulse (”Red 3PPE”) is present near the coupling slit, where the field of the pump pulse is strong, but its photoemission yield is small due to the higher-order photoemission process needed to overcome the work function. In the range of spatiotemporal separation (*x* ≳ 40 μm), it is negligible. Real space plots of *Y*_static_ are available in the Supporting Information.

The resulting dynamic photoemission yield *Y*_source_(*x*, *y*, *E*, Δ*t*) is shown in Figure 2B as a 2D projection to the *x*–Δ*t* - plane. The integration range of *y* = 55–79 μm corresponds to the irradiated section of the coupling slit. The integration range in energy of *E* – *E*_F_ = 1.45–1.55 eV corresponds to electrons excited from close to the Fermi edge. For these highest available excitation energies, the inelastic lifetime of hot electrons is shortest, in the order of tens of femtoseconds,^39^ and the trace is the least broadened in the time domain. Therefore, this range is best suited for comparison with Figure 2A to assign regions of predominant photonic and plasmonic excitation for the analysis of the respective electron energy distributions.

The obtained hot electron trace in Figure 2B can be clearly attributed to the spatiotemporal characteristics of the source: After an intense nonpropagating feature that is caused by the pump pulse, a trace of electrons excited by the SPP is observed. For larger delays and distances from the coupling slit, the photon- and plasmon-induced hot electrons are clearly separated. The electrons observed in the propagating trace after the pump pulse has faded can be assigned to the photoemission channel in which the SPP contributes the initial excitation (green highlighted path in Figure 1E). This signal serves as an observable for the plasmon-induced hot electrons which are the main focus of this study.

Comparing the experimental PEEM trace to the temporal profile of the calculated energy density rate (Figure 2B vs 2A), the influence of the inelastic electron lifetime is visible in the slower temporal attenuation of the PEEM signal. An additional effect of broadening is added by the finite temporal length of the probe pulse, which universally provides a lower limit to the temporal width of a pump–probe signal, given by the cross-correlation between the two pulses.

**Figure 3:**
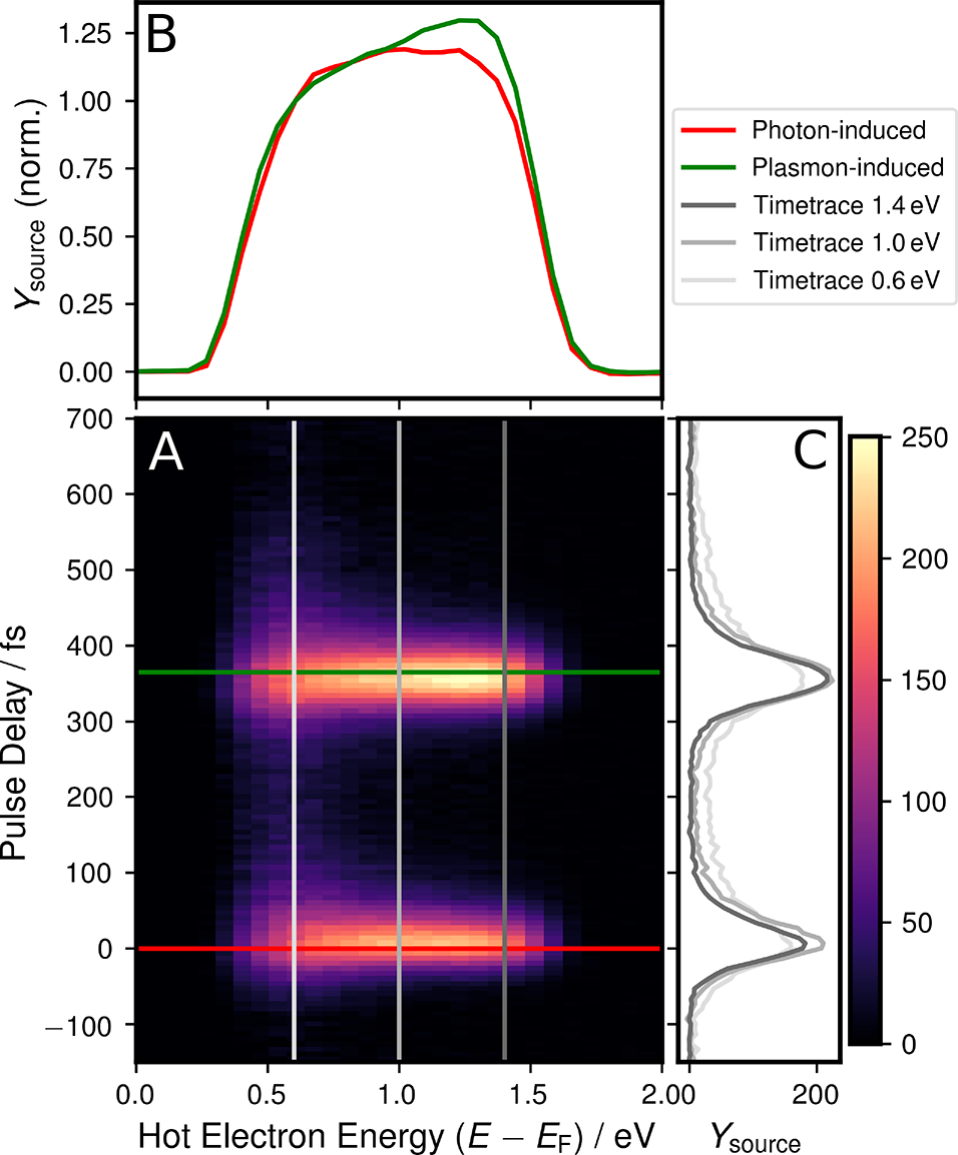
(A) Projection of the 4D-data set of photoemission yield for *x* = 90–100 μm, *y* = 55–79 μm to the time-energy plane. (B) Spectra of photon-induced (red line) and plasmon-induced (green line) hot electrons, extracted along the respective lines in (A) at Δ*t*_0_ = 0 fs (red) and Δ*t*_SPP_ = 365 fs (green) and normalized to the onset of the low-energy cutoff at *E* – *E*_F_ = 0.6 eV. (C) Time trace of electrons emitted from excitation energies of *E* – *E*_F_ = 1.4, 1.0, and 0.6 eV, extracted as cuts along the respective gray lines in (A). The colorbar and *Y*_source_ axis values are given in counts.

For the study of plasmon-induced hot electrons, and in contrast to previously reported two-color PEEM experiments with SPP,^57^ the key in our isolation scheme is the combination of information on all available dimensions, in our case time, space, and energy. In the full 4-dimensional data set, different aspects of spectral and lifetime information can be extracted by evaluating different regions of the hypercube. This can be seen in the projection of certain slices of the data set: In the range of *x* = 95 μm, where the photon and plasmon contributions are most clearly separated in space and time (white shaded range in Figure 2B), a projection of the experimental 4D data set to the *E*–Δ*t*-plane was plotted in Figure 3A. Line cuts were extracted using a temporal integration range of ±25 fs and an energy integration range of ±0.05 eV. These cuts represent electron spectra for specific times (green, red, Figure 3B) and time traces for constant energies (gray, Figure 3C), respectively.

In the time traces (in Figure 3C), the typical lifetime behavior of hot electrons, governed by the Fermi liquid theory,^39^ is visible: The inelastic lifetimes *T*_1_ increase with smaller excitation energy, which leads to more electrons remaining for later times after excitation (lighter gray compared to darker gray graphs).

In the energy domain, a photon-induced and a plasmon-induced hot electron spectrum is plotted for delay times of Δ*t*_0_ = 0 fs (red) and Δ*t*_SPP_ = 365 fs (green), representing the stages at which the respective photonic and plasmonic contributions to the source field are most dominant. The spectra in Figure 3B are normalized to their low-energy cutoff (for absolute values see raw spectra in the Supporting Information). A comparison shows that the higher excitation energies appear stronger in the plasmon-induced spectrum (see also difference spectrum in the Supporting Information). This apparent preference for higher-energetic excitation is intriguing: Given that the photonic signal follows the familiar excitation mechanism with the energy distribution governed by the DOS of the material, it suggests that for the plasmonic signal a different excitation mechanism is involved. The high-energy feature emerging among the still present, continuous spectrum of photon-induced electrons hints that both the familiar single-particle excitation and a different plasmonic excitation might contribute. This would reflect the hybrid nature of the surface plasmon polariton as an electromagnetic wave (*polariton* aspect) bound to a collective charge motion (*plasmon* aspect). Following this reasoning, the *polariton* aspect would cause an Einsteinian photoexcitation known from light, while the *plasmon* aspect selectively excites electrons close to *E*_F_, similar to the effect known from bulk plasmons.^18−23^

An influence of the weaker blue–red channel should manifest in the time-resolved signal predominantly at time steps where the blue pulse precedes the red, for Δ*t* ≲ Δ*t*_0_ or Δ*t* ≲ Δ*t*_SPP_, owing to the order of the involved excitation processes. Especially in the lower energy channels, secondary electrons would be expected due to the shorter lifetime of the intermediate state as discussed above. Such an influence is not observed in the electron traces, which leads us to conclude that the channel is not of significant strength, confirming that the observed hot electron distributions are predominantly excited by the pump and SPP pulses.

### Momentum Microscopy

To gain further insight into the intriguing feature of high-energy preference of SPP-induced hot electrons, the momentum microscopy mode of the PEEM^58−61^ was used to characterize the distribution of plasmon-induced hot electrons in energy and momentum space (*k*-space).

To use the concept of spatiotemporal separation in this mode, the probe laser focus was positioned in the center of the field-of-view, ∼100 μm to the right of the coupling slit, and an iris aperture in the image plane of the electron-optical column was closed to limit the detection area to a diameter of ∼30 μm in real space. In this way, the SPP pulse propagates through the selected area after the pump pulse is no longer present on the sample, similar to the temporal evolution in the white-shaded area of Figure 2 B.

The momentum space distribution of the emitted electrons was detected by imaging the back-focal plane of the objective lens of the PEEM to the detector, thus acquiring a *k*-space photoemission data set of the shape *Y*(*k*_*x*_, *k*_*y*_, *E*, Δ*t*). Similarly to the subtraction of the static yield in eq 3, the signal contribution caused by the SPP source field (green highlighted path in Figure 1E) was extracted using the delay time when the SPP pulse is centered on the selected area, Δ*t*_SPP_ = 360 fs, and subtracting the static yield before the temporal overlap at Δ*t*_static_ = −334 fs:
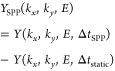
4

A reference momentum distribution for photon-induced electrons *Y*_photon_ (gray in red–blue channel in Figure 1E) was measured with the red pump laser also centered to the field-of-view instead of focused on the excitation slit, using identical sample position and PEEM settings. The pump-induced signal contribution was extracted at time zero Δ*t*_0_ = 0 fs, subtracting the static probe yield in the same way:
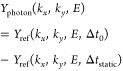
5where *Y*_ref_ is the photoelectron yield of the reference measurement.

**Figure 4:**
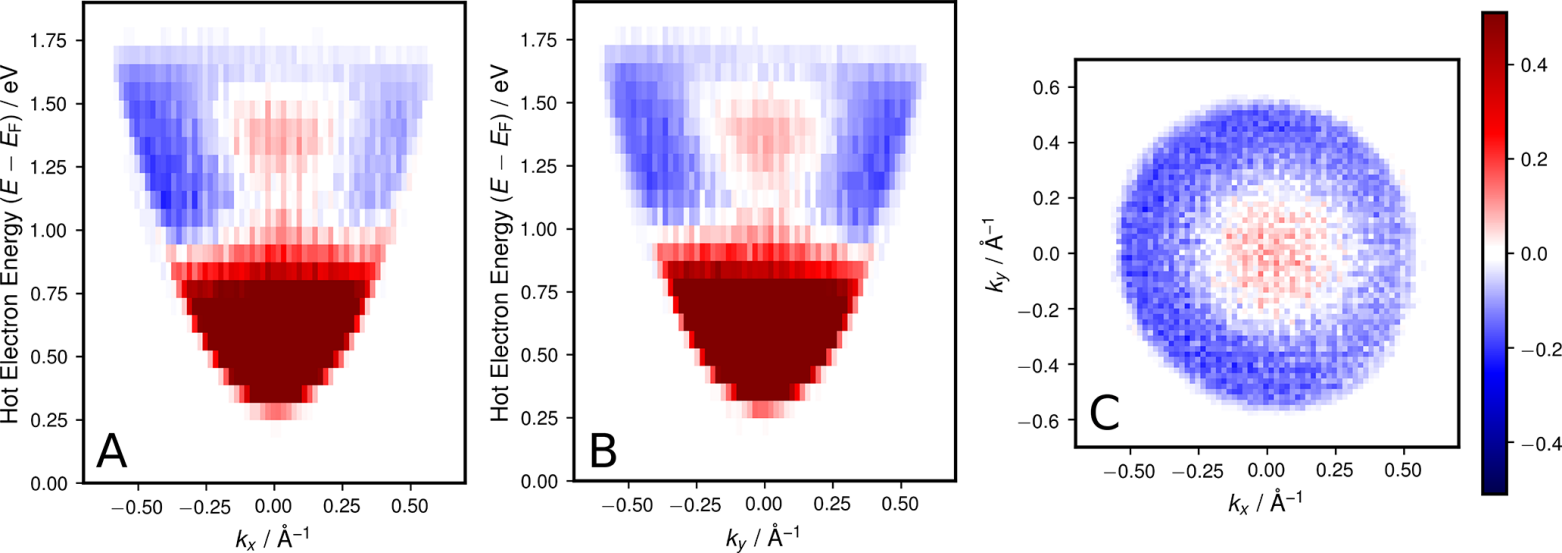
Momentum distribution of plasmon-induced hot electrons relative to photon-induced hot electrons. Cuts of the normalized difference values Δ*Y*(*k*_*x*_, *k*_*y*_, *E*) are shown along (A) the *E*–*k*_*x*_-direction for *k*_*y*_ = 0 Å^–1^, (B) the *E*–*k*_*y*_-direction for *k*_*x*_ = 0 Å^–1^, and (C) the *k*_*x*_–*k*_*y*_-direction for *E* – *E*_F_ = 1.4–1.5 eV.

Figure 4 shows the normalized difference Δ*Y*(*k*_*x*_, *k*_*y*_, *E*) between plasmon-induced and photon-induced hot electrons in momentum space. The values were normalized by
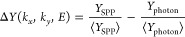
6where ⟨*Y*_SPP_⟩ and ⟨*Y*_photon_⟩ are the respective mean values of *Y*_SPP_ and *Y*_photon_ over the full energy and momentum range, to account for the arbitrary difference in absolute signal strength. Plots of the same *k*-space cuts of the individual yields *Y*_SPP_ and *Y*_photon_ are provided in the Supporting Information.

The high-energy feature described in the previous section is present in the *k*-space data in Figure 4 as a contiguous red region in the energy range 1.2–1.55 eV in the center of the momentum range, close to the Γ̅-point of the surface. In this region, the value of Δ*Y*(*k*_*x*_, *k*_*y*_, *E*) is positive, corresponding to an enhanced generation of hot electrons by plasmons with respect to photons.

From the usual perspective of surface science, where one typically explains features in *k*-space in terms of the electronic band structure, this feature is surprising: Due to the polycrystalline nature of the sample and the consequent randomness of crystallite orientations contained in the detection area, band structure effects should average out and one expects a homogeneous momentum distribution of electrons.

Interestingly, no significant anisotropy or asymmetry of the plasmon-induced electrons along the propagation direction of the SPP (*k*_*x*_-direction) is observed. The enhancement is concentrated at small values of the in-plane momenta *k*_∥_ = (*k*_*x*_^2^+*k*_*y*_^2^)^1/2^, but otherwise symmetric in *k*-space. This implies that the wave vector orientation and related momentum of the SPP wave seems to have no relevant influence.

The obtained momentum distribution could be an important clue for a different mechanism taking part in electron excitation by surface plasmons. The transfer of energy from the collective to single-particle excitation seems to efficiently couple to electrons with low momentum and close to the Fermi surface. Possible origins of this phenomenon are to be looked for in the nature of the SPP: Apart from the difference in the orientation of oscillating electric and magnetic fields, the movement of electrons as part of the collective excitation (*plasmon* aspect) in contrast to the single-particle case could be crucial. But ultimately, at this point, a conclusive explanation of the plasmon decay mechanism, which links the collective motion to the single particle momentum, is missing.

In Figure 4A,B, which show the normalized difference in the energy vs momentum distribution in both directions (*E*–*k*_*x*_-plot and *E*–*k*_*y*_-plot), a strong enhancement for *smaller* energies of *E* – *E*_F_ < 0.8 eV is also apparent, which seems to contradict the result of the real space experiment. It turns out though, that this is an artifact of the measurement parameters: In contrast to the pump pulse, which impinges on the sample under near-normal incidence and is present only for the laser pulse duration, the SPP pulse propagates with finite velocity through the detection area selected by the iris aperture. This results in an apparent broadening of the SPP pulse duration, increasing the detection probability for cascade electrons, which are produced by inelastic decay after the initial excitation from the SPP pulse. We show in the Supporting Information that this effect can be mitigated using a smaller aperture size and extracting the data from time windows of the full time-resolved measurement, selected to compensate for the apparent broadening. Unfortunately, the use of a very small aperture causes a different artifact in the electron-optics; therefore, we chose to show the present data in the main manuscript.

## Conclusion

To summarize, we have shown how direct experimental access to plasmon-induced hot electrons was realized in a photoemission experiment. The results show a preference for higher-energetic excitations with small in-plane momentum induced by surface plasmons in contrast to photons, independent of the crystal orientation of the metal surface. This suggests that the mechanism of plasmon-enhanced energy harvesting is more fundamentally linked to the nature of the surface plasmon and its decay into hot carriers, rather than just a simple field-enhancement at the metallic surface. At this point it is unclear if the observed effect is of related physical origin as the similar-looking effect known for bulk plasmons.^18−23^ The aspect of a collective oscillation of electrons in both bulk and surface plasmons leads to the hypothesis that a similar mechanism may be at play. On the contrary, it must be noted that many aspects are fundamentally different: While the bulk plasmon is in its nature a resonance effect of the bulk electronic system, the SPP case constitutes a decay of surface plasmons at the metal–dielectric interface and far from resonance. In both cases, the preferential excitation of electrons at *E*_F_ is evident, but as the SPP follows the frequency of the pump light rather than being pinned to a resonance frequency, the non-Einsteinian characteristic of subsequent pinning of electron energy off from *ℏω* of the driving laser is *not* to be expected for the SPP case.

For the future, this poses the important question whether similar decay mechanisms also occur for LSP and therefore plasmons of all types. Although LSP and SPP are different with respect to their defined resonances and localization as opposed to continuous dispersion and propagation, the two are closely related. This can be seen in cases where localized modes are created by a superposition of guided SPP, for example, in whispering gallery resonators.^62^ Therefore, it seems likely that the underlying mechanism for SPP also contributes to electron excitation from LSP, although it might be harder to resolve experimentally as discussed above. Further theoretical studies will be needed to provide a unified picture of plasmonic decay channels.

Irrespective of fundamental origin, a preferential generation of high-energy electrons holds great potential for chemical and energy harvesting purposes. Unlike the typical continuous distribution of energy to electrons and holes, here, we have a case in which a concentration of energy to ”hot” electrons with only low-energetic (“cold”) holes is evident, which could be of key advantage. For technical applications, the most promising decay processes are charge-transfer excitations such as chemical interface damping^17,24,63^ at a metal interface, where carriers are directly excited in the adjacent material. It is yet to be determined whether the observed effect could play a role in this context, but an enhancement of plasmonic excitation for electrons at *E*_F_ was also reported for plasmonic nanoparticle interfaces.^25−27^ On the contrary, the electronic structure at the chemical interface in such systems additionally provides emerging dephasing pathways, which are of importance for plasmon decay,^17,64^ as well as the dynamics of hot electrons at the interface.^65^ In fact, emergent plasmonic excitation features were explained in terms of interface effects in most previous work. The concept of spatiotemporal separation will bring further insight into the multitude of influential effects for plasmon damping and plasmonic excitation at such interfaces. But, even in the demonstrated case of a bare metal surface, the presented measurement scheme may further contribute to a more quantitative understanding of the fundamental properties of plasmon-induced hot carriers in metals. Future steps could include, for instance, a systematic variation of the photon energy as well as intensity of the exciting light pulses. Complementary, systematic differences in the energy and momentum dependent quasiparticle lifetime of these hot carriers could be uncovered by this scheme in combination with the recently introduced approach of time-resolved two-photon momentum microscopy.^60^

## Methods

### Time-Dependent Energy Density Calculations

A detailed description of the calculation method is given in our previous work.^56^ In our simulations, we consider a Gaussian pulse profile for the incoming laser field, including all components of electric and magnetic fields. The system consists of the gold sample filling the half-space *z* ≤ 0 and a perfect vacuum filling the other half-space *z* > 0. As a spatially defined coupling structure providing SPP, we used a step edge perpendicular to the interface of height 50 nm at *x* = 0 μm irradiated at normal incidence.

The laser parameters applied in the simulations are a laser wavelength centered at λ = 800 nm, a pulse duration of τ = 23 fs, a Gaussian width of 25 μm centered at *x* = 10 μm away from the position of the step edge, and a pump pulse energy of 1 nJ. The pulse arrives at the sample surface centered with its maximal amplitude at *t* = 0 fs. The coupling parameter at the step edge, β, is set to 0.2. It describes the ratio of moduli of the SPP magnetic field amplitude and the incident laser magnetic field amplitude at the origin of the step edge (*x* = 0). The obtained energy density rate was averaged over one full period of the oscillating fields to account for the lack of phase resolution of the experiment.

We represent the dielectric half-space by a perfect vacuum with ϵ_d_ ≡ 1. The metallic half-space is modeled with the dielectric function reported by Olmon *et al*.^66^ for evaporated gold.

### Sample

The sample was produced in the Nano Structuring Center (NSC) at TU Kaiserslautern. A layer of gold with a thickness of 250 nm was sputter-deposited onto a substrate cut from a native-oxidized Silicon wafer. The straight coupling slit with a width of ∼100 nm, a depth of ∼230 nm, and a length of 80 μm was etched by focused ion beam (FIB) milling. A submonolayer of Cesium was evaporated onto the sample surface *in situ* to reduce the work function for the PEEM measurement. Further details and images are available in the Supporting Information.

### Two-Color Pump–Probe Setup

A femtosecond Ti:sapphire laser (Spectra Physics Tsunami) with an average output power of ca. 700 mW produces pulses at a central wavelength of λ = 800 nm with a pulse duration down to τ_pump_ = 23 fs and a repetition rate of 75 MHz. In a home-built two-color time-resolved optical setup, after using a prism compressor for dispersion compensation, the pulses are split up in two arms of an interferometer. In one arm, the (”probe”) pulse is frequency-doubled by second harmonic generation (SHG) through a β barium borate (BBO) crystal and compressed again with another prism pair; then, it is routed over a linear delay stage to adjust the delay between the pump and probe. The ”pump” arm passes a fixed optical route of equal effective length. The two arms are subsequently recombined and irradiated onto the sample in the PEEM setup.

The first prism unit is optimized for the shortest-possible pump pulse on the sample. This unavoidably leads to a chirped red pulse in the BBO and reduces the effective spectral width of the SHG. The chirp of the blue probe pulse is subsequently compensated by the second prism unit for the shortest possible duration on the sample, but due to the fundamental limit given by the time-bandwidth-product, it is longer than the pump pulse. During the optimization of the prism compression unit, it was approximated to τ_probe_ ≲ 61 fs with a crosscorrelation measurement in near-threshold two-color photoemission and disregarding lifetime effects.

### Photoemission Electron Microscopy

We used a customized commercial PEEM setup (IS-PEEM, Focus GmbH), which is designed for laser irradiation under near-normal incidence.^45^ The laser beam is routed over a small mirror, introduced into the electron column of the microscope near the optical axis, which leads to an angle of incidence AOI ≈ 4°.

The beam diameters on the sample are approximately 50 μm, with the pump beam centered at the excitation slit at *x* = 0 μm. The probe beam was offset to *x* ≈ 70 μm for the real space experiments for sufficient probe intensity throughout the field-of-view and to *x* ≈ 100 μm for the momentum microscopy experiments for optimal alignment in the center of the selected sample area.

The photoelectrons were detected with a delayline detector (DLD), which records their positions as well as their time-of-flight, with reference to a trigger signal from the laser. Thus, the kinetic energy of each photoelectron was measured through proper energy-calibration of the detector’s time channels, and a time- and energy-resolved PEEM experiment^67^ was performed. The PEEM can be operated as a momentum microscope by imaging the back-focal plane of the objective lens, in this way detecting the angular distribution of photoelectrons.

In the real space experiment, the SPP trace was observed through the probe pulse, which hits the sample under a near-normal but not perfectly normal incidence of AOI ≈ 4°. Therefore, the in-plane component of the wave vector of the probe pulse *k*_∥_ = −ω/*c*_0_ · sin(AOI) contributes to the perceived propagation. For the dashed white line in Figure 2B, this was taken into account by plotting the perceived SPP velocity (see the derivation in the Supporting Information)
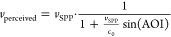
7which is slightly slower than the real SPP velocity *v*_SPP_.

### Data Evaluation

For all tr-PEEM data sets, several runs of exposures for the same list of pump–probe delays were acquired and summed up during postprocessing to improve the data quality.

The energy axis offset of all data sets was calibrated to the excited electron energy by fitting a Fermi distribution to the high-energy cutoff of the photoelectron spectrum acquired with the blue probe laser. The fitted Fermi energy was set to *E* – *E*_F_ = 3.1 eV according to the photon energy of the laser. The low-energy cutoff, caused by the work function of the material, is visible in Figure 3B as a slope in the range *E* – *E*_F_ = 0.3–0.6 eV. The latter value, where the photonic excitation spectrum converges to its flat top, was chosen for normalization.

The real space data in Figures 2B and 3 were binned in groups of (2, 16) pixels in (*x*, *y*), and a constant background across energy channels was subtracted for each binned pixel prior to the subtraction of the static signal in eq 3 to increase the signal-to-noise ratio.

The *k*-scale in the momentum space measurements (Figure 4) was calibrated by fitting a parabolic free-electron dispersion to the low-energy cutoff of the data in *E*–*k*_*x*_- and *E*–*k*_*y*_-cuts of the center of the data.

For the calculation in eq 6, the measured reference data was slightly shifted by (−0.5, 2.0, 0.15) pixels in (*k*_*x*_, *k*_*y*_, *E*) to correct for small differences in alignment. To reduce noise, a Gaussian filter with a kernel (sigma) of (0.005 Å^–1^, 0.005 Å^–1^, 0.2 energy channels) in (*k*_*x*_, *k*_*y*_, *E*) was applied, and then, both data sets were binned in groups of (8, 8) pixels in (*k*_*x*_, *k*_*y*_). To select only the region of statistically relevant data, voxels with less than 80 counts in *Y*_SPP_ or *Y*_photon_ were ignored.

## Supporting Information Available

Figures of SEM images, real space PEEM images, plots of the individual source term components, raw electron spectra, difference spectrum, propagation dependence of the SPP-induced electron energy distribution, individual momentum microscopy data of the photon and SPP contributions, and additional momentum microscopy experimental results with mitigation of the secondary electron artifact and discussions of sample fabrication and characterization details and derivation of the observed SPP velocity (PDF)
Movie of animated spatiotemporal separation scheme (AVI)
Movie of spatiotemporal dynamics plasmon-induced hot carriers (AVI)

## Supplementary Material

nn1c06586_si_001.pdf

nn1c06586_si_002.avi

nn1c06586_si_003.avi
