# Supplementary material for: Energy and Momentum Distribution of Surface Plasmon-Induced Hot Carriers Isolated via Spatiotemporal Separation
Source: ACS Nano. 2021 Dec 1;15(12):19559–69. doi: 10.1021/acsnano.1c06586 (PMC8717854; doi:10.1021/acsnano.1c06586)
Supplement: Supplementary file 1 — nn1c06586_si_001.pdf [file nn1c06586_si_001.pdf]

Energy and Momentum Distribution of Surface Plasmon-Induced Hot Carriers Isolated *via* Spatiotemporal Separation

Supplementary Information

Michael Hartelt,[1,*] Pavel N. Terekhin,[1] Tobias Eul,[1] Anna-Katharina Mahro,[1] Benjamin Frisch,[1] Eva Prinz,[1] Baerbel Rethfeld,[1] Benjamin Stadtmüller,[1,2] and Martin Aeschlimann[1]

[1]*Department of Physics and Research Center OPTIMAS, TU Kaiserslautern, Erwin-Schrödinger-Straße 46, 67663 Kaiserslautern, Germany*

[2]*Institute of Physics, Johannes Gutenberg University Mainz, Staudingerweg 7, 55128 Mainz, Germany*

(Dated: October 20, 2021)

I. SAMPLE FABRICATION AND CHARACTERIZATION

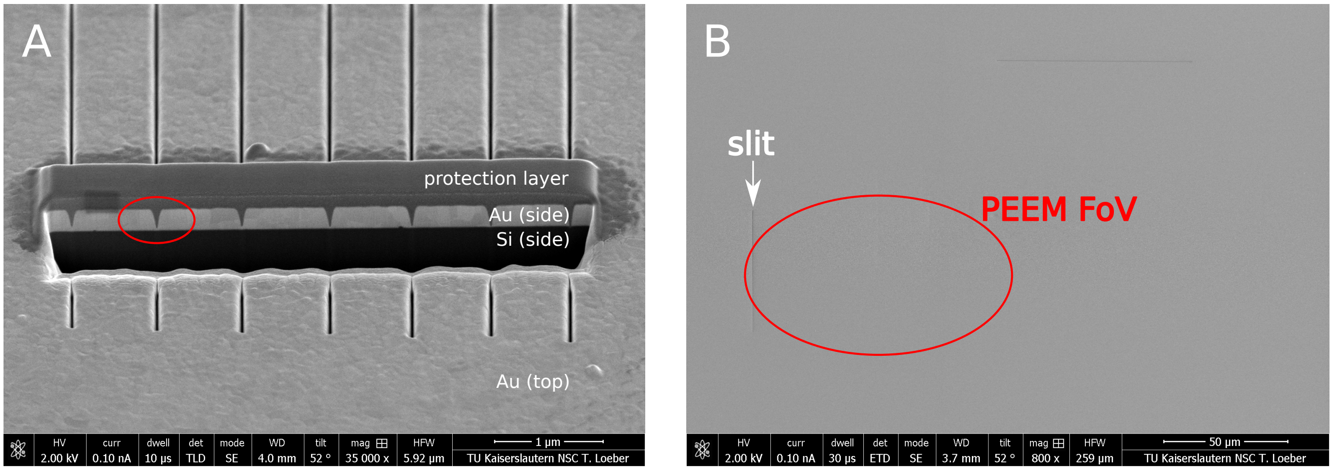

Figure S1. SEM images of the sample, taken with the electron column of the combined FIB/SEM setup under an angle of 52° to the surface normal during sample fabrication. A) Side view of the excitation slit, taken during a dose test. The slit circled in red corresponds to the selected dose for the structures used in the PEEM measurements. B) Overview of the sample area used in the PEEM experiments. The red circle corresponds to the approximate field-of-view of the real-space PEEM measurement. Note that the actual field-of-view is circular, which appears as an elliptical shape due to the image tilt of the SEM. The horizontal slit to the upper right of the image was milled for reference, but *not* used in the PEEM measurements. Both slits shown in the image have a length of 80 µm. SEM images by Thomas H. Loeber, NSC, TU Kaiserslautern, used with permission.

The sample was fabricated in the Nano Structuring Center (NSC) at TU Kaiserslautern. A sputter deposition system (Oerlikon UNIVEX 450 C) was used to deposit a layer of gold with a thickness of 250 nm onto a substrate cut from a native-oxidized Silicon wafer. The excitation slits were milled with a combined focussed ion beam (FIB) and scanning electron microscope (SEM) setup (FEI Helios Nanolab 650). The quality of the fabrication was optimized by writing test structures with different ion doses, depositing a protection layer locally onto them using Ion Assisted Chemical Vapor Deposition (IACVD), and cutting a straight vertical profile, imaged from the side with SEM in Figure S1A. From the image, a rough estimation of the characteristic grain size of the polycrystalline Au film can be made to the range of 100 nm to 500 nm, seen most clearly in the shading at the Au side view surface. The chosen parameters resulted in slits with a width of $\sim$100 nm at the gold surface and a depth of $\sim$230 nm in the shape of a "V-grove". These parameters were used to mill the straight slits of length 80 µm, shown in Figure S1B. Subsequently, the sample was transferred to the ultra-high vacuum (UHV) system of the PEEM setup and a sub-monolayer of Cesium was evaporated onto the sample surface *in situ*. In this way, the work function of the Au surface was reduced to $\Phi \approx 3.4\,\mathrm{eV}$ to maximize the energy range of the obtained hot electron spectrum without reaching one-photon photoemission (1PPE) by the probe laser. The achieved spectral range, corresponding to this value, confirms that in the measurement both contributions from the pump and probe pulses in the "Red-Blue 2PPE" signal work in the linear range. In the static "Blue-Blue 2PPE" signal, the probe pulse contributes with quadratic order, accordingly.

II. REAL SPACE PEEM IMAGES

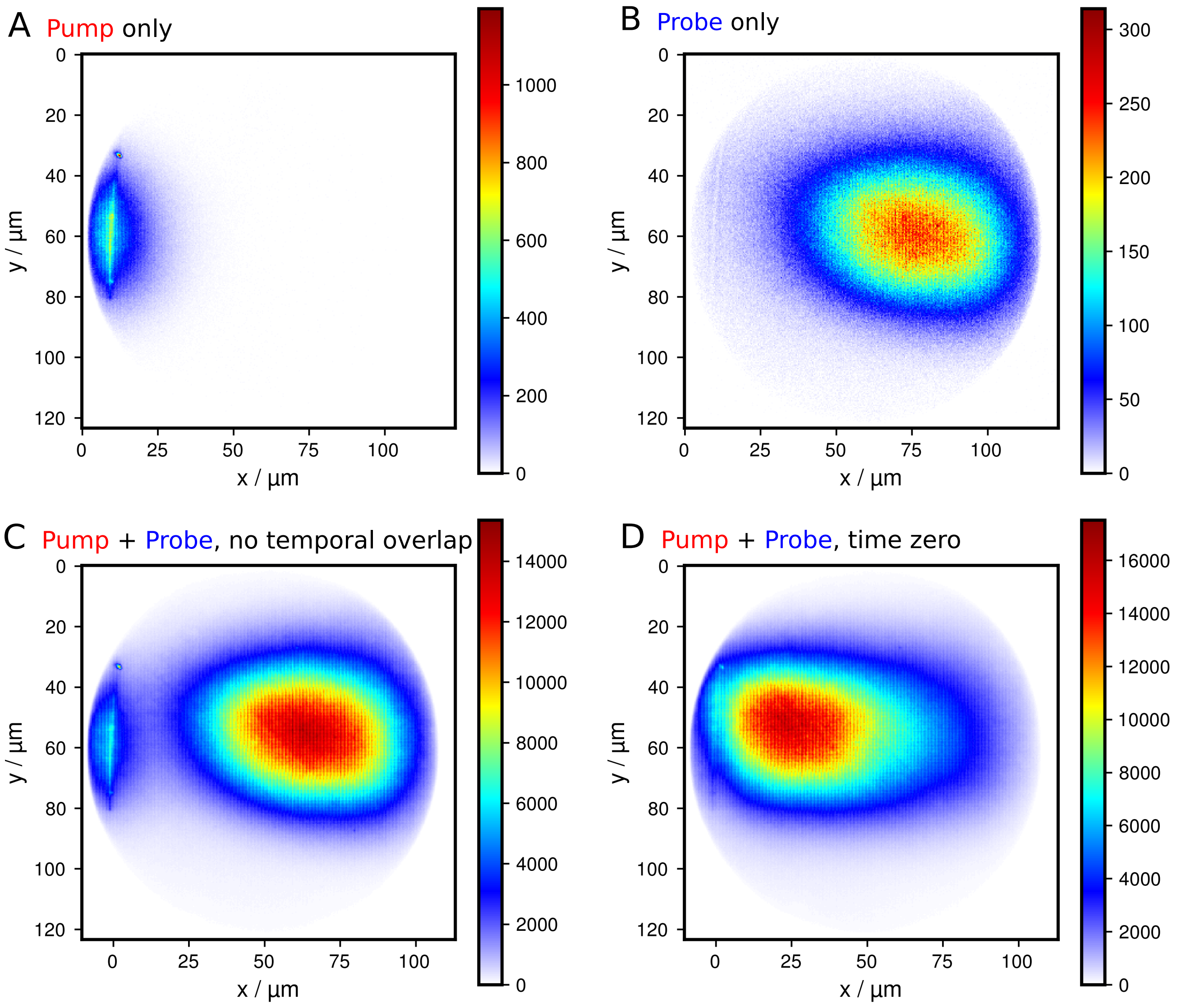

Figure S2. Real space PEEM images of the sample, with identical laser alignment and PEEM settings, shown in raw electron counts without any normalization, integrated over all electron energy channels. Note that the absolute magnitudes of the count values are *not* comparable, due to different exposure times and temporal integration ranges, respectively. Therefore, independent color bars were used for optimal contrast. A) PEEM image for a static exposure with only the red pump laser. The excitation slit at $x = 0\,\mu\text{m}$ is illuminated by the pump pulse. The imaged photoelectrons are emitted in a three-photon photoemission process ("Red-Red-Red 3PPE"). B) PEEM image for a static exposure with only the blue probe laser. Mainly visible is the spatial profile of the probe pulse. The imaged photoelectrons are emitted in a two-photon photoemission process ("Blue-Blue 2PPE"). C) PEEM image from the full 4D dataset for an integration range of $\Delta t = -300\,\text{fs}$ to $-200\,\text{fs}$ (no temporal overlap). This corresponds to the static probe contribution Y_{probe}, integrated over all electron energies. Both channels from A and B are present, but they contribute separately, because the two pulses are not present on the sample at the same time. The probe contribution dominates due to its lower photoemission order. D) PEEM image from the full 4D dataset for an integration range of $\Delta t = -10\,\text{fs}$ to $10\,\text{fs}$ (time zero). Compared to C, the enhanced yield between the coupling edge and the probe pulse is caused by electrons excited by the pump pulse and photoemitted by the probe pulse ("Red-Blue 2PPE").

III. COMPONENTS OF THE THEORETICAL SOURCE TERM

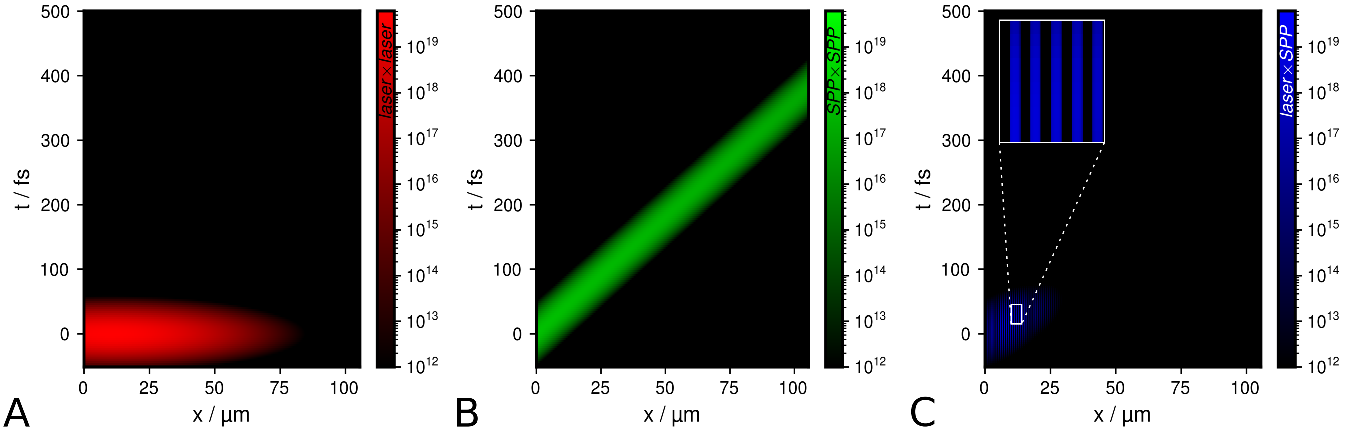

Figure S3. Calculation of the source term components A) *laser-laser*, B) *SPP-SPP*, and C) *laser-SPP*. The time-dependent energy density of the electromagnetic field contributions at the surface is shown for an excitation pulse with $\lambda = 800\,\mathrm{nm}$ and a pulse duration of 23 fs. The pulse maximum arrives at the sample surface at $t = 0\,\mathrm{fs}$. The colorbars are given in $\mathrm{J\,s^{-1}\,m^{-3}}$. The inset in C) is a zoom of $x = 10\,\mu\mathrm{m}$ to $14\,\mu\mathrm{m}$ and $t = 15\,\mathrm{fs}$ to $45\,\mathrm{fs}$, showing the striped interference pattern.

IV. CALCULATION OF THE OBSERVED SPP VELOCITY

The real part of the SPP wave vector k'_{SPP} from the solution of the wave equation at the interface [S1] is given by

$$k'_{\mathrm{SPP}} = \Re\left(\frac{\omega}{c_0} \cdot \sqrt{\frac{\epsilon_{\mathrm{m}}\epsilon_{\mathrm{d}}}{\epsilon_{\mathrm{m}} + \epsilon_{\mathrm{d}}}}\right), \tag{S1}$$

while the probe pulse counter-propagates with an in-plane wave vector of

$$k_{\parallel} = -k_{\mathrm{probe}} \sin(\mathrm{AOI}) = -\frac{\omega}{c_0} \sin(\mathrm{AOI}), \tag{S2}$$

the negative sign signifies the orientation of the projected wave vector in negative x-direction.

This results in the well-known effect for SPP waves observed in PEEM, [S2] the so-called beating pattern

$$k_{\mathrm{perceived}} = k'_{\mathrm{SPP}} - k_{\parallel}. \tag{S3}$$

We can link the perceived SPP group velocity $v_{\mathrm{perceived}}$ and the actual SPP group velocity v_{SPP} by taking the derivative over the frequency ω from equation S3:

$$\frac{1}{v_{\mathrm{perceived}}} = \frac{dk_{\mathrm{perceived}}}{d\omega} = \frac{dk'_{\mathrm{SPP}}}{d\omega} - \frac{dk_{\parallel}}{d\omega} = \frac{1}{v_{\mathrm{SPP}}} + \frac{\sin(\mathrm{AOI})}{c_0}. \tag{S4}$$

Solving for $v_{\mathrm{perceived}}$ yields the relation used in the main manuscript:

$$v_{\mathrm{perceived}} = v_{\mathrm{SPP}} \cdot \frac{1}{1 + \frac{v_{\mathrm{SPP}}}{c_0} \sin(\mathrm{AOI})}. \tag{S5}$$

V. RAW SPECTRA AND DIFFERENCE SPECTRUM

The photon-induced and plasmon-induced hot electron spectra from the Δt-Energy-plot in the main manuscript are normalized to their work function edges at the low-energy cutoff for $E - E_F = 0.6\,\text{eV}$. For reference, the raw spectra without normalization are given in Figure S4. The difference in absolute height is caused by the strength of the respective source field.

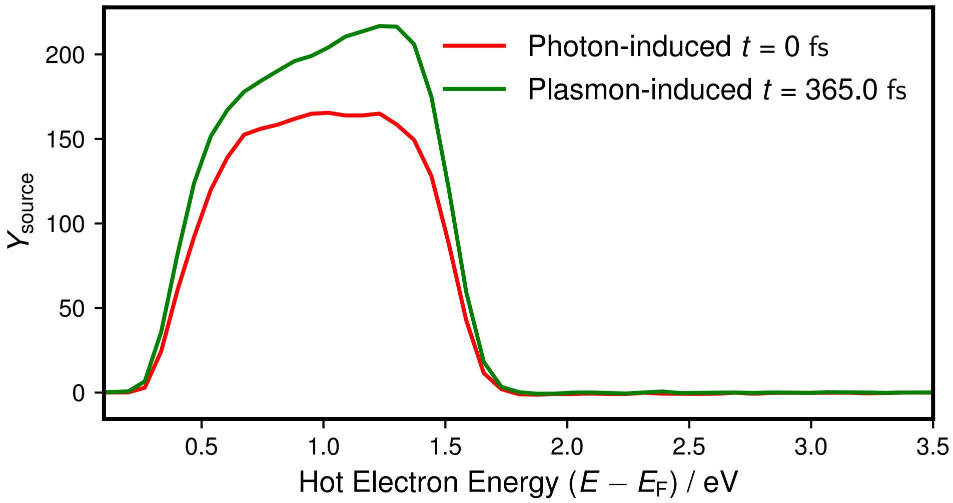

Figure S4. Raw Spectra from the Δt-Energy-plot in the main manuscript, without normalization. The Y_{source} values are given in counts.

The two normalized curves from the main manuscript were subtracted for Figure S5. The main feature is the plasmonic excitation peak at high energies. According to the reasoning in the main manuscript, this would correspond to the electron spectrum of the *plasmon* aspect of SPP. The smaller peak in the low-energy cutoff region, representing a slight difference in the shape of the work function edge, is most likely caused by the SPP propagation through the finite real space integration area. As discussed in Section VIII this causes an artifact of increased detection of cascade electrons.

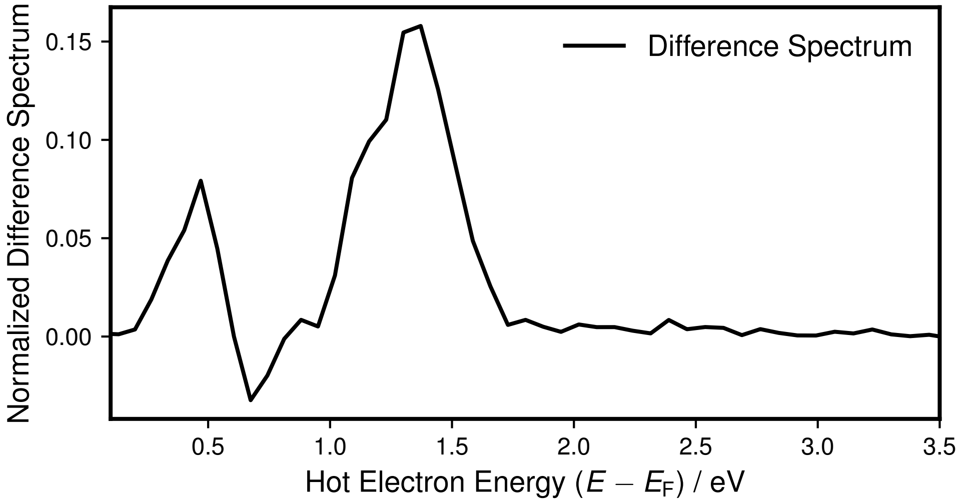

Figure S5. Difference Spectrum between $Y_{\text{source}}(\Delta t_{\text{SPP}})$ and $Y_{\text{source}}(\Delta t_0)$ as normalized in the main manuscript.

VI. PROPAGATION DEPENDENCE OF THE ENERGY DISTRIBUTION

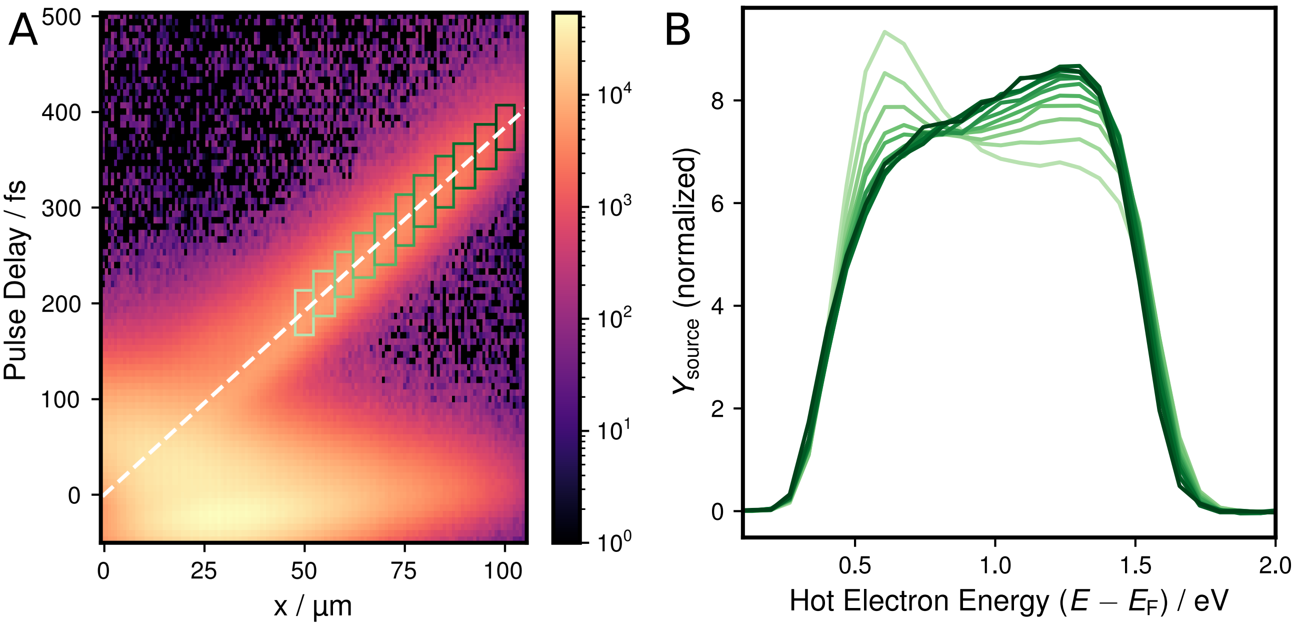

Figure S6. Propagation dependence of the plasmon-induced hot electron spectrum. A) Selection of regions along the SPP propagation in the x-t-plot as in the main manuscript. B) Plasmon-induced hot electron spectra, evaluated from the regions in A, marked in the respective color. Each curve was normalized by its mean value for comparability.

The plasmon-induced hot electron spectrum was evaluated at different positions along the propagation trace of the SPP pulse to identify an influence of the SPP propagation. Selections, centered along the propagation line $x = v_{\mathrm{perceived}}\ \Delta t$, with a size of 50 fs in Δt and 5 µm in x (approximated to the nearest voxel in the dataset), were evaluated. In the earlier spectra (lighter green), an influence of remaining secondary electrons from excitations from the pump pulse is present (incomplete spatiotemporal separation). This shows in a peak in the low-energy range of the spectrum. Later on, when the time separation is sufficient for these secondary electrons to decay, the spectra converge to the plasmon-induced spectrum as shown in the main manuscript and in Figure S4 (darker green). For the range of sufficient spatiotemporal separation, no significant influence of the SPP propagation is observed.

VII. MOMENTUM MICROSCOPY DATA

The momentum microscopy plots in figures S7, S8, and S9 were shifted, noise-filtered and binned as described under Data Evaluation in the Methods section in the main manuscript.

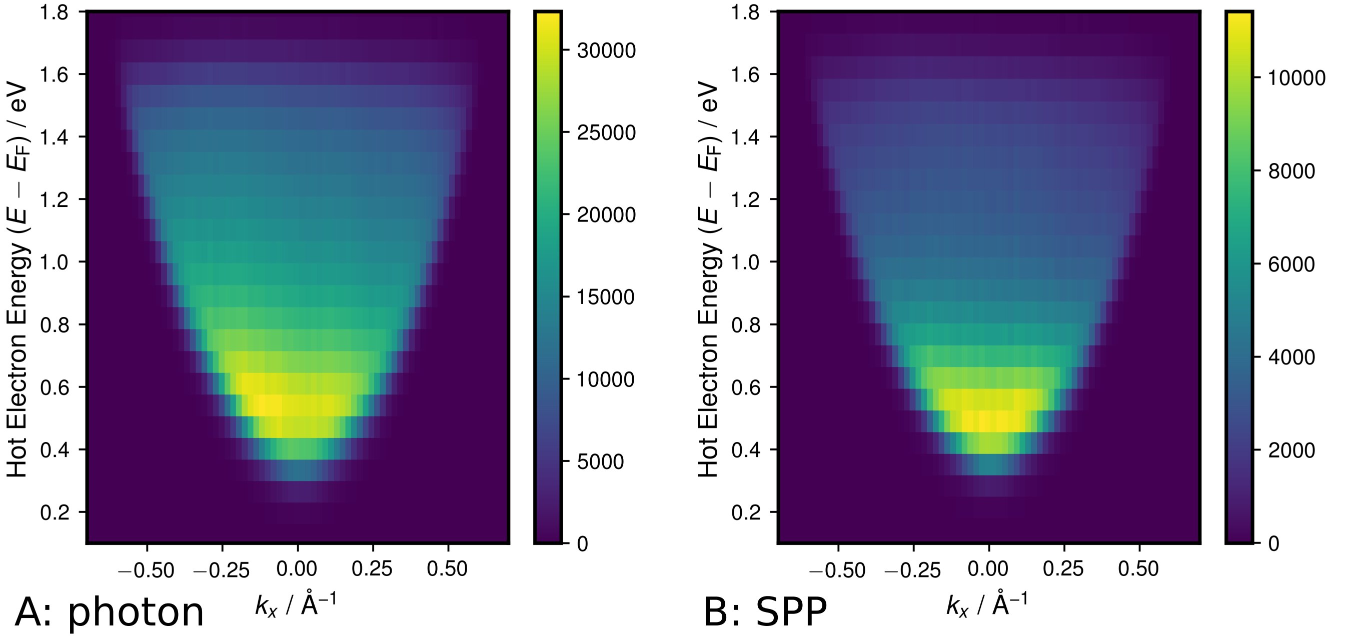

Figure S7. Momentum microscopy data A) $Y_{\text{photon}}(k_x, k_y, E)$ and B) $Y_{\text{SPP}}(k_x, k_y, E)$ along the E-k_x-direction for $k_y = 0\,\text{Å}^{-1}$.

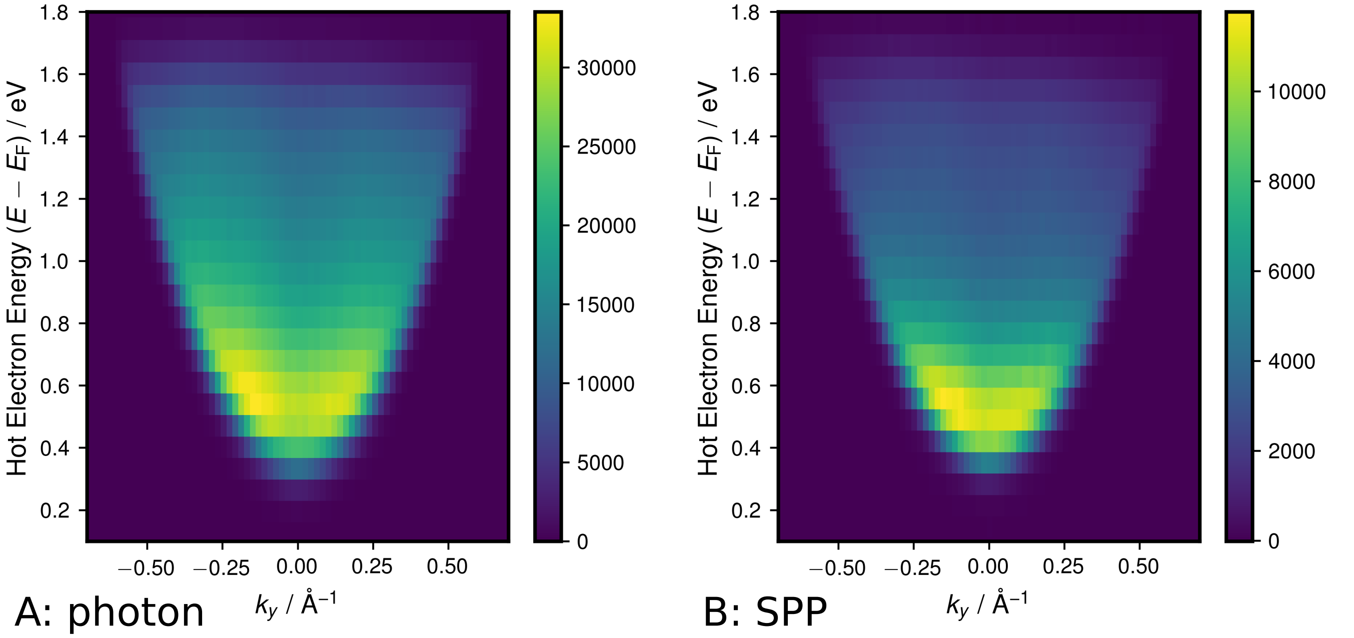

Figure S8. Momentum microscopy data A) $Y_{\text{photon}}(k_x, k_y, E)$ and B) $Y_{\text{SPP}}(k_x, k_y, E)$ along the E-k_y-direction for $k_x = 0\,\text{Å}^{-1}$.

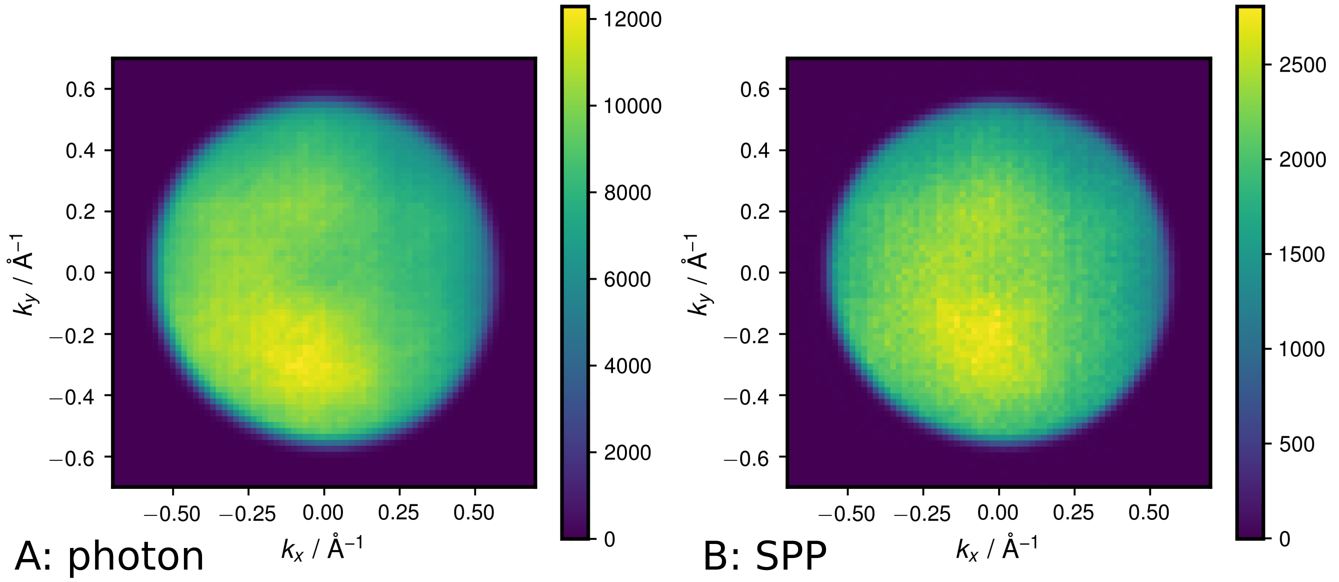

Figure S9. Momentum microscopy data A) $Y_{\text{photon}}(k_x, k_y, E)$ and B) $Y_{\text{SPP}}(k_x, k_y, E)$ along the k_x-k_y-direction for $E - E_{\text{F}}$ =1.4 eV to 1.5 eV.

VIII. COMPENSATION OF THE SPP PROPAGATION ARTIFACT IN THE MOMENTUM DISTRIBUTION

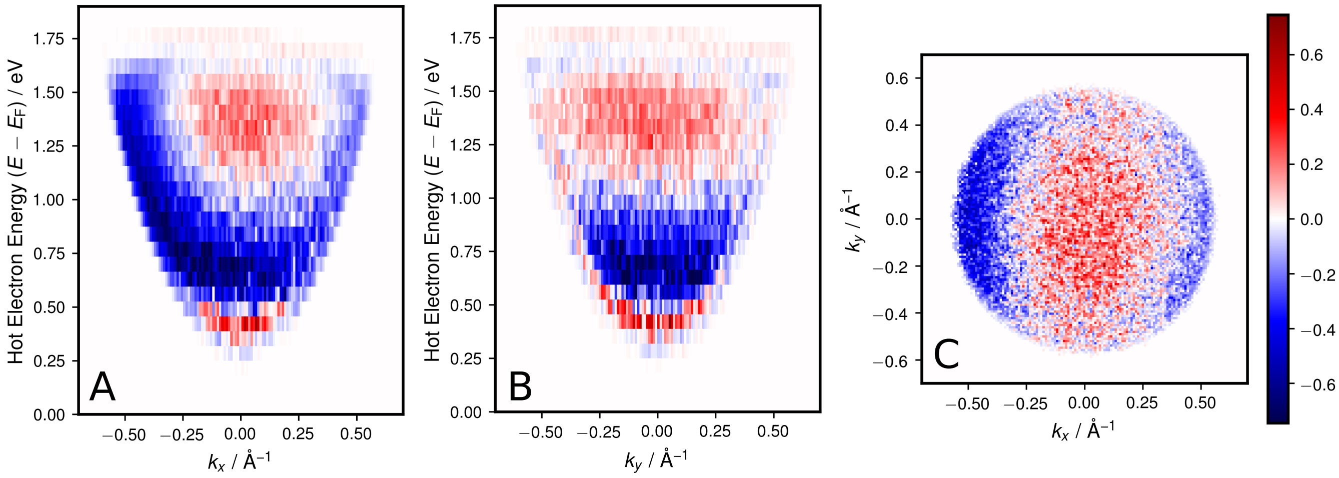

Figure S10. Momentum distribution of plasmon-induced hot electrons relative to photon-induced hot electrons, mitigating the apparent broadening effect by the propagation of the SPP pulse through the field-of-view. Plotted are cuts of the normalized difference values $\Delta Y(k_x, k_y, E)$ along A) the E-k_x-direction for $k_y = 0\,\text{Å}^{-1}$, B) the E-k_y-direction for $k_x = 0\,\text{Å}^{-1}$, and C) the k_x-k_y-direction for $E - E_\text{F}$ =1.4 eV to 1.5 eV.

As described in the main manuscript, the apparent low-energy enhancement of plasmon-induced electrons in the k-space data is caused by an apparent broadening of the SPP pulse duration due to its propagation through the detection area. This is an observation artifact caused by the experimental scheme and setup, and not to be confused with a real, dispersive pulse broadening. (Due to the very linear SPP dispersion over the used wavelength range, the dispersive broadening during the propagation time is small.) To mitigate this effect, a measurement with reduced aperture size of ≈18 µm was performed, recording the full time trace of $\Delta t = -334$ fs to 800 fs. The evaluation was carried out as described in the main manuscript, but using integration ranges instead of fixed values for the respective delay times: $\Delta t_\text{SPP} = 345$ fs to 355 fs, $\Delta t_\text{photon} = -80$ fs to 80 fs, and $\Delta t_\text{probe} = -334$ fs to -167 fs. In this way, a prolonged time window for the photonic signal compensates for the propagation time of the SPP pulse through the detection area. To select only the region of statistically relevant data, voxels with less than 80 counts in Y_SPP or less than 5 counts in Y_photon were ignored.

The result in Figure S10 shows that the larger integration range for the photonic signal compensates for the propagation artifact and the signal enhancement caused by cascade electrons is mitigated. The high-energy feature remains, but it is deformed along the k_y-direction. This deformation is caused by an electron-optical artifact when the iris aperture is set to very small diameters. It is then no longer symmetric, but a hexagonal shape elongated along the y-direction.

IX. MOVIES

- Animation of the Scheme of Spatiotemporal Separation
  `spatiotemporal_scheme_animated.avi`
- Movie of the Spatiotemporal Dynamics Plasmon-induced Hot Carriers. Shown is $Y_\text{source}(x, y, E, \Delta t)$ for an electron energy of $E = 1.5$ eV. The field-of-view and axes are as depicted in Figure S2.
  `timeseries_binned_fermi.avi`

REFERENCES

* hartelt@physik.uni-kl.de
[S1] Maier, S. A. *Plasmonics: Fundamentals and Applications*; Springer Science & Business Media: New York, NY, USA, 2007.

[S2] Kubo, A.; Pontius, N.; Petek, H. Femtosecond Microscopy of Surface Plasmon Polariton Wave Packet Evolution at the Silver/Vacuum Interface. *Nano Letters* **2007**, *7*, 470–475.
